# Association of cartilage metabolism biomarkers and 25(OH)D levels with muscle biomechanical functions in professional rowers and canoeists

**DOI:** 10.1038/s41598-024-51272-8

**Published:** 2024-01-11

**Authors:** Małgorzata Ogurkowska, Tomasz Podgórski, Alicja Nowak

**Affiliations:** 1 Department of Biomechanics, Poznan University of Physical Education, Królowej Jadwigi Street 27/39, 61-871 Poznań, Poland; 2 Department of Physiology and Biochemistry, Poznan University of Physical Education, Poznań, Poland

**Keywords:** Biochemistry, Biophysics, Biomarkers

## Abstract

The purpose of the study was to assess the association of cartilage metabolism biomarkers and vitamin D metabolite levels with muscle biomechanical functions in professional rowers and canoeists. The serum levels of aggrecan, cartilage oligomeric matrix protein (COMP), and 25-hydroxyvitamin D (25(OH)D) were determined in elite male sweep-oar rowers (n = 24) and canoeists (n = 15). This was followed by a biomechanical study consisting in isometric measurement of peak torque (PT) of muscles involved in the rowing cycle in the athletes. There were found significant correlations of COMP with the ratio of trunk PT flexor to extensor (*p* < 0.05) and 25(OH)D with trunk PT—left rotators (*p* < 0.05), knee joints PT—left and right flexor (*p* ≤ 0.01), ratio of knee joint PT—right flexor to knee joint PT—right extensor (p < 0.05) in rowers and aggreccan with elbow joint PT of the right flexor (*p* ≤ 0.01) and extensor (*p* = 0.05) in canoeists. The correlations of COMP and aggrecan levels with PT of the muscle groups studied in rowers and canoeists indicate the importance of stabilizing the muscular system in cartilage metabolism. The relationship between 25(OH)D status and biomechanical parameters confirm that vitamin D plays an important role in maintaining skeletal muscle health.

## Introduction

Rowing and canoeing are popular endurance and strength sports with considerable training loads in which the technique of rowing depends mainly on the athlete’s preferences^1^. Continuous refinement of a specific form of movement, whose aim is to optimise and perfect the execution of the pattern, cause considerable mechanical loads in these athletes, thereby possibly inducing a process that may lead to the damage of articular cartilage and development of osteoarthritis (OA)^2,3^.

There are biochemical methods which may be used to diagnose the risk of cartilage destruction. Biochemical markers may help to observe abnormalities in the turnover of articular cartilage and synovial tissues and they are helpful tools in early diagnosis before irreversible damage has occurred. Several authors have suggested that aggrecan and cartilage oligomeric matrix protein (COMP) can be non-invasive and sensitive biomarkers in detecting subtle changes in cartilage and synovial tissues^4–6^. COMP is a non-collagenous protein isolated from cartilage and it is also present in other tissues in much lower amounts. Its primary role is to maintain the stability of the collagen II network^6^. Aggrecan participates in articular cartilage degradation and protection which are upregulated by mediators associated with joint inflammation and overloading^7,8^.

Physical activity with different types of mechanical loading induces diverse articular cartilage responses and changes in the serum levels of cartilage markers^4,6,8^. In the review, Roberts et al. presented the current understanding of joint serum biomarker adaptation to mechanical loading, both under acute and chronic training loads. However, with regard to research on athletes, studies were mainly concerned with cyclists, swimmers, runners, volleyball and soccer players^8,9^. We found only one study comparing cartilage and bone degradation markers in rowers to other athletes and a non-athletic group. The authors showed that the group of rowers and runners had higher levels of cartilage remodelling or degradation comparing to swimmers and the control group^10^. Moreover, using magnetic resonance imaging (MRI) method, Bittersohl et al. observed signs of hip cartilage degeneration comparing results to controls (asymptomatic volunteers of similar ages who were not competitive rowers)^2^.

The biomechanics of rowing differs considerably between rowers and canoeists. When analysing the movement of a rower, attention should be paid to the sequence of movements performed by the body parts involved^11,12^. The correct sequence of movements, i.e. engaging the lower limbs, followed by the torso and then arms, can lead to more efficient rowing and therefore translate to a higher average boat speed^13,14^. Engaging the torso too early is one of the most common mistakes made by athletes. It contributes to the development of overloads in the lumbar spine^15–17^. However the propulsive force of the boat in canoeists results from the forward motion of the hips and torso followed by the pull of the oar. In the case of canoeists, however, the whole body is involved in the oar stroke^18^. This is a positive aspect, as the loads are distributed over different joints, instead of being accumulated in the lumbar spine, as it is the case with rowers.

It could be interesting to observe the relationship between muscle biomechanical functions and serum biochemical markers of cartilage metabolism in elite rowers and canoeists because muscle strength and the flexor-to-extensor strength ratio play an important role in joint stability and in the prevention of joint injuries^3^. We found no studies simultaneously evaluating the results of biomechanical measurements and biochemical markers of cartilage metabolism in these athletes. In view of the foregoing, the aim of the study was to assess the relationship of serum concentrations of aggrecan and COMP in rowers and canoeists with muscle biomechanical functions and to compare the levels of the mentioned markers between the athlete groups. The levels of 25-hydroxyvitamin D (calcidiol, 25(OH)D), a vitamin D metabolite, were additionally assessed due to the involvement of this vitamin in modulating muscle strength and function^19^.

## Results

Table 1 presents the basic somatic characteristics, biochemical indices and biomechanical parameters with an explanation of their abbreviations for rowers and canoeists. The comparative analysis showed that the rowers, as compared to the canoeists, were characterised by a significantly higher body height (*p* ≤ 0.01), body mass (*p* ≤ 0.01) and higher serum concentrations of COMP (*p* < 0.05) and 25(OH)D (*p* < 0.05).

**Table 1:** The basic somatic characteristics, biochemical indices and biomechanical parameters in groups of rowers (n = 24) and canoeists (n = 15) and results of comparative analysis between groups.

Parameters	Rowers (n = 24)	Canoeists (n = 15)	*p*-value
Age (yrs)	22.29(3.89); 21.0(20.0–23.0)	21.13(2.72); 22.0(19.0–22.0)	0.773^b^
Train. experience (yrs)	9.0(3.44); 8.0(7.0–9.5)	9.27(3.63); 8.0(6.0–12.0)	0.908^b^
Body height (m)	1.93(0.06); 1.9(1.9–1.9)	1.82(0.06); 1.78(1.78–1.84)	< 0.001^a^
Body mass (kg)	94.82(8.94); 93.6(88.8–102.1)	81.93(9.93); 79.3(74.7–90.4)	< 0.001^a^
COMP (ng/ml)	648.96(198.24); 611.9(478.2–794.6)	495.28(146.47); 504.3(341.0–608.7)	0.014^a^
Aggrecan (μg/ml)	2.86(0.53); 2.7(2.5–3.0)	3.16(0.51); 3.16(2.59–3.65)	0.089^b^
25(OH)D (ng/ml)	30.01(6.10); 29.7(25.4–34.3)	25.33(5.79); 24.0(20.2–30.7)	0.023^a^
HJPT-LE (Nm/kg)	5.05(0.90); 5.0(4.4–5.7)	5.31(1.22); 5.4(4.5–5.9)	0.447^a^
HJPT-LF (Nm/kg)	1.65(0.33); 1.6(1.4–1.8)	2.0(0.50); 1.9(1.7–2.1)	0.013^a^
TPT-LR (Nm/kg)	1.12(0.26); 1.1(0.9–1.3)	1.47(0.28); 1.5(1.3–1.6)	< 0.001^a^
TPT-LR (Nm/kg)	1.77(0.43); 1.8(1.6–1.8)	2.06(0.48); 2.1(1.6–2.6)	0.091^b^
TPT-RR (Nm/kg)	1.12(0.26);1.1(0.8–1.3)	1.38(0.31); 1.3(1.1–1.7)	0.007^a^
TPT-E (Nm/kg)	6.25(0.79); 6.3(5.7–6.8)	7.30(1.21); 7.1(6.9–7.6)	0.002^a^
TPT-F (Nm/kg)	3.57(0.42); 3.5(3.3–3.8)	3.54(0.50); 3.4(3.1–3.9)	0.854^a^
EJPT-RF (Nm/kg)	1.13(0.21); 1.1(0.9–1.3)	1.14(0.25); 1.2(1.0–1.3)	0.893^a^
EJPT-LF (Nm/kg)	1.02(0.19); 1.0(0.9–1.1)	1.26(0.30); 1.2(1.2–1.4)	0.004^a^
EJPT-LE (Nm/kg)	0.69(0.11); 0.6(0.6–0.7)	0.81(0.11); 0.8(0.7–0.9)	0.001^b^
EJPT-RE (Nm/kg)	0.64(0.18); 0.6(0.5–0.7)	0.76(0.16); 0.7(0.6–0.9)	0.027^b^
KJPT-LF (Nm/kg)	1.93(0.35); 1.9 (1.7–2.3)	2.04(0.44); 1.9(1.7–2.3)	0.409^a^
KJPT—RF (Nm/kg)	3.17 (0.66); 3.2(2.7–3.8)	3.60(0.69); 3.5(3.2–4.1)	0.062^a^
KJPT—LE (Nm/kg)	1.97(0.36); 1.9(1.7–2.3)	1.96(0.45); 1.9(1.7–2.1)	0.942^a^
SJPT-RE (Nm/kg)	1.46(0.27); 1.4(1.3–1.6)	1.52(0.40); 1.4(1.2–1.9)	0.626^a^
GSLL-A-LE (N/kg)	26.30(4.78); 25.5(22.6–30.4)	29.37(5.61); 27.6(25.0–32.8)	0.076^a^
GSLL-S-LE (N/kg)	26.95(6.30); 25.4(22.5–29.7)	30.97(5.27); 32.0(27.5–34.0)	0.046^a^

Results are expressed as mean (SD); median (interquartile range); ^a^*T*-test; ^b^*U-Mann–Whitney* test.

COMP, cartilage oligomeric matrix protein; GSLL-A-LE, global strength of lower limb – asymmetric technical—left extensors; GSLL-A-RE, global strength of lower limb—asymmetric technical—right extensors; GSLL-S-LE, global strength of lower limb—symmetric technical—left extensors; HJPT-LF, hip joint peak torque—left flexor; HJPT-LE, hip joint peak torque—left extensor; KJPT-LE, knee joint peak torque—left extensor; KJPT-LF, knee joint peak torque—left flexor; KJPT-RF, knee joint peak torque—right flexor; SJPT-RE, shoulder joint peak torque—right extensor; EJPT-LE, elbow joint peak torque—left extensor; EJPT-RE, elbow joint peak torque—right extensor; EJPT-LF, elbow joint peak torque—left flexor; EJPT-RF, elbow joint peak torque—right flexor; TPT-E, trunk peak torque—extensors; TPT-F, trunk peak torque—flexors; TPT-LR, trunk peak torque—left rotators; TPT-RR, trunk peak torque—right rotators; RKJ-R, ratio of KJPT-RF/KJPT-RE; RHJ-L, ratio of HJPT-LF/HJPT-LE; RHJ-R, ratio of HJPT-RF/HJPT-RE; RSJ-L, ratio of SJPT-LF/ SJPT-LE; REJ-R, ratio of EJPT-RF/EJPT-RE; RT, ratio of TPT-F/TPT-E.

Given to the differences in somatic characteristics noted between the groups of rowers and canoeists, interactions between these groups of athletes (between discipline, body mass and body height) were performed for the COMP and aggrecan variables using an equal slopes model. It was found that the interactions in both cases, i.e. for COMP and aggrecan concentrations, were not statistically significant (*p* = 0.569 and *p* = 0.649, respectively) and therefore there was no basis for performing an analysis of differences for these variables between the groups of rowers and canoeists after excluding the effects of body mass and height.

With regard to the biomechanical indices (Table 1), significantly higher peak torque of trunk extensors (TPT-E, *p* ≤ 0.01), peak torque of trunk left rotators (TPT-LR, *p* < 0.05), peak torque of trunk right rotators (TPT-RR, *p* ≤ 0.01) were recorded in the group of canoeists. In contrast, the ratio of peak torque of trunk flexors to peak torque of trunk extensors (RT) was significantly (*p* = 0.0023) higher in rowers. A comparative analysis of lower limb joints between the groups of athletes showed a tendency towards higher global strength of lower limb—asymmetric technical—left extensors (GSLL-A-LE) in canoeists; measurement of the global strength of lower limb—symmetric technical—left extensors (GSLL-S-LE) showed the difference to be significant (*p *< 0.05). Analysis of the PT values of the hip joint muscles showed significantly higher values for the peak torque of hip joint left flexor (HJPT-LF, *p* < 0.05) in canoeists. Comparison of the upper limb joint muscles showed that peak torque of elbow joint left flexor (EJPT-LF), peak torque of elbow joint left extensor (EJPT-LE) and peak torque of elbow joint right extensor (EJPT-RE) were significantly higher in canoeists than in rowers (*p* ≤ 0.01, *p* ≤ 0.01 and *p* < 0.05, respectively). In the right limb, significantly higher PT values were also found in canoeists, but only for the extensor muscle. At the same time, the ratio of EJPT-RF/EJPT-RE (REJ-R) differed significantly between the disciplines, with a higher value in rowers (*p* = 0.0025). Comparison of the shoulder joint between the groups of athletes showed that there was only a tendency towards a higher ratio of SJPT-LF/ SJPT-LE (RSJ-L) in canoeists.

**Figure 1:**
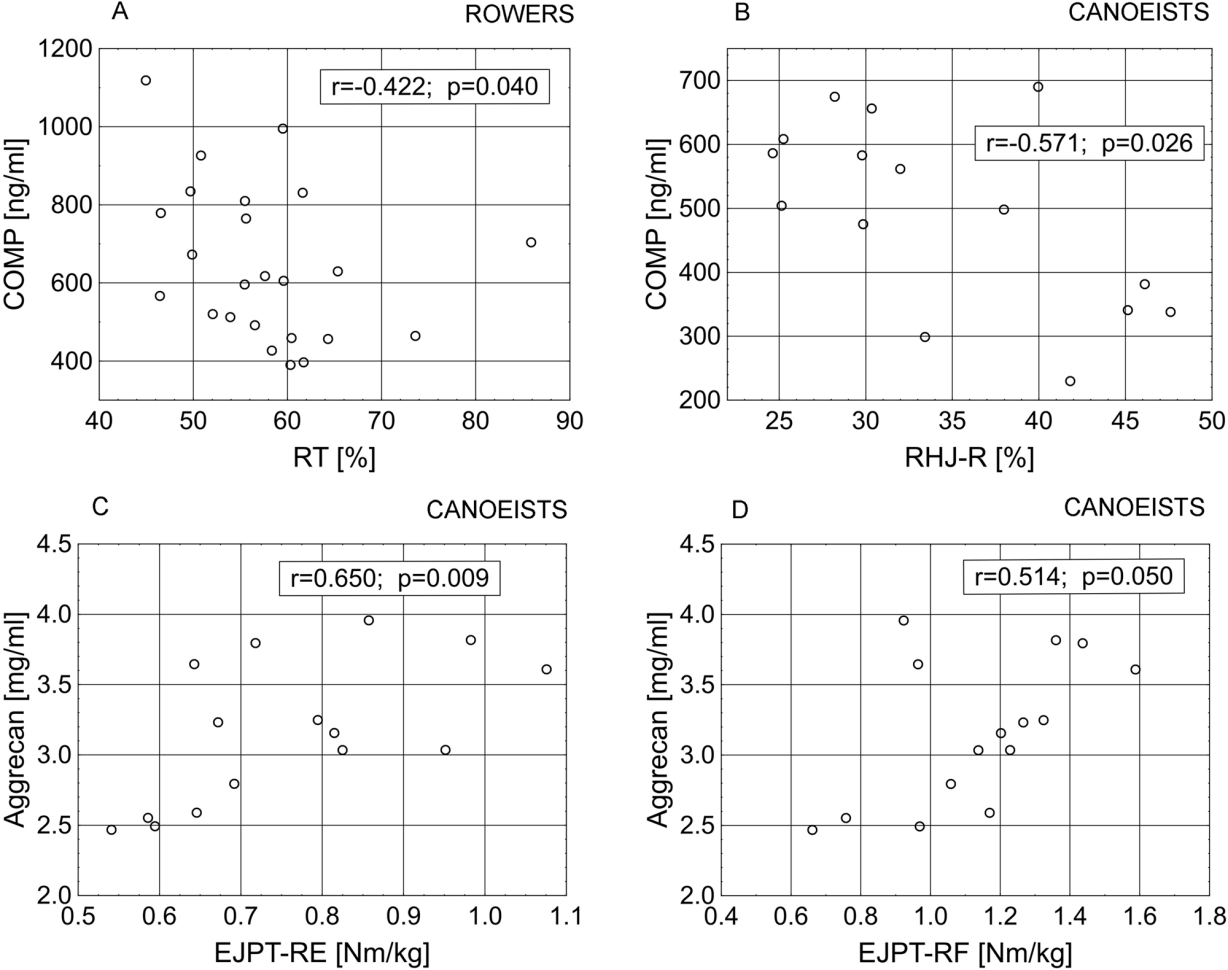
Relationships between serum concentration of cartilage metabolism/degradation markers and biomechanical muscle functions in rowers and canoeists. EJPT-RE, elbow joint peak torque—right extensor; EJPT-RF, elbow joint peak torque—right flexor; RHJ-R, ratio of HJPT-RF/HJPT-RE; RT, ratio of TPT-F/TPT-E.

**Figure 2:**
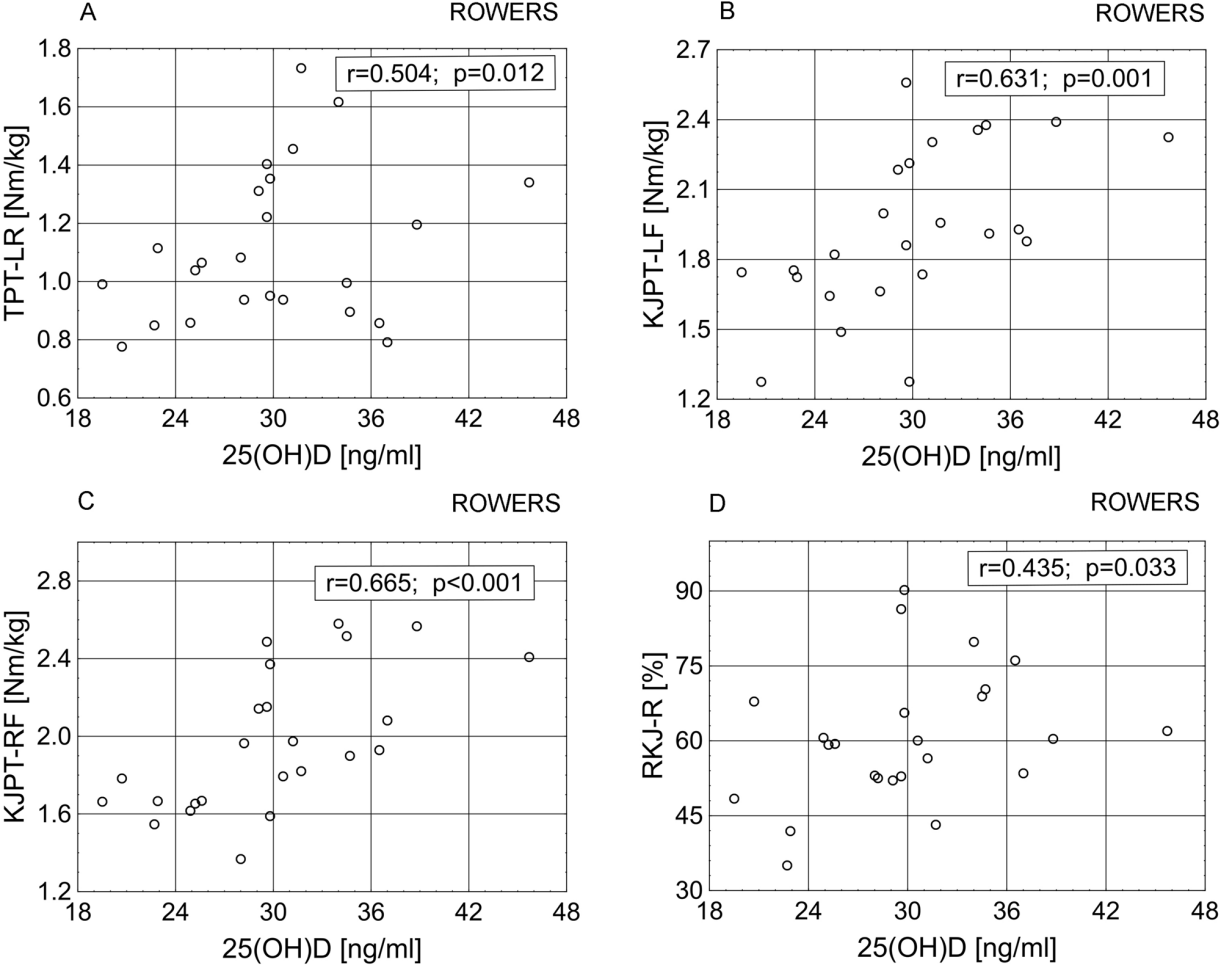
Relationships between biomechanical muscle functions and serum concentration of 25(OH)D in rowers. KJPT-LF, knee joint peak torque—left flexor; KJPT-RF, knee joint peak torque—right flexor; TPT-LR, trunk peak torque—left rotators; RKJ-R, ratio of KJPT-RF/KJPT-RE.

In the group of rowers there were found:

- a significant negative correlation of COMP with RT (Fig. 1A, p < 0.05) and a tendency with the ratio of hip joint peak torque—left flexor to hip joint peak torque—left extensor (RHJ-L) (r = − 0.388, *p* = 0.061), and a tendency to positive relationships with the global strength of the lower limb—asymmetric technical—right extensors (GSLL-A-RE) (r = 0.380, *p* = 0.067) and hip joint peak torque—left extensor (HJPT-LE) (r = 0.363, *p* = 0.082);
- a tendency towards significant positive correlations of the serum concentration of aggrecan with TPT-E (r = 0.374, *p* = 0.097), EJPT-LF (r = 0.398, *p* = 0.054);
- significant positive correlations of serum 25(OH)D concentration with TPT-LR, knee joint peak torque—left flexor (KJPT-LF), knee joint peak torque—right flexor (KJPT-RF), ratio of KJPT-RF/KJPT-RE (RKJ-R) (Fig. 2A,B,C,D, respectively) and the tendency towards significance of the relationship with trunk peak torque—right rotators (TPT-RR) (r = 0.369, *p* = 0.076).

In the group of canoeists there were found:

- a significant negative correlation of COMP concentration with the ratio of HJPT-RF/HJPT-RE (RHJ-R) (Fig. 1B) and tendency with RHJ-L (r = − 0.500, *p* = 0.058), TPT-RR (r = − 0.507, *p* = 0.054), GSLL-A-LE (r = − 0.450, *p* = 0.092), EJPT-LE (r = -0.504, *p* = 0.056); positive correlations of aggrecan concentration with EJPT-RE, elbow joint peak torque—right flexor (EJPT-RF) (Fig. 1C,D, respectively) and tendency with EJPT-LF (r = 0.471, *p* = 0.076);
- a tendency towards significance of the relationships between 25(OH)D concentration and the RT (r = -0.462, *p* = 0.083), EJPT-RE (r = 0.458, *p* = 0.086), shoulder joint peak torque—right extensor (SJPT-RE) (r = − 0.458, *p* = 0.086).

Significant relationships were found between COMP and body height for the group of all athletes (n = 39, r = 0.374, *p* = 0.019); correlations of 25(OH)D concentration with height and body mass (r = 0.765, *p* < 0.001 and r = 0.585, *p* = 0.022, respectively) and between training experience and the concentration of COMP for canoeists (r = 0.527, *p* = 0.044).

## Discussion

The main findings of this study were a higher serum concentration of the articular cartilage metabolism/degradation marker − COMP in rowers as compared to canoeists and relationships in both groups between the aforementioned marker and biomechanical parameters of muscles as active stabilisers of the human musculoskeletal system.

Roberts et al.^8^ concluded that prolonged exercise may result in higher levels of serum COMP which can remain elevated for several hours to days. It is speculated that the serum COMP concentrations in the athletes participating in the study may be indicative of the condition of articular cartilage and its response to joint loading during training, which differs significantly in terms of movement characteristics in rowing and canoeing. The higher COMP concentration in rowers compared to canoeists reflects a higher level of cartilage metabolism/degradation, which may result from both a direct response of the articular cartilage to the mechanical stimulus associated with previous training loads and chronic effects of training on the state of this tissue. It should be emphasised that there are significant differences in joint loading during performance of the rowing movement in the sports in question. The efficiency of rowing depends mainly on the rower's ability to develop a push-off force (lower limbs), which is further transmitted through the torso and upper limbs to the oar. This creates the propulsive force of the boat^20,21^. The rower, by rapidly straightening the knee joints during early drive phase, develops a very strong force, referred to as leg drive. Thus, the rowing movement primarily involves knee extensors, which should perform most of the work in the pull phase with the simultaneous torso extension. However, rowers making technical errors, which are often due to insufficient strength of knee extensors^22^, use the erector spinae, which contributes to its stiffening over time and, as a consequence, development of the pathomechanism of overload changes within lumbar tissues^23^. Moreover, the movement is asymmetric, as long-oar rowers row with two hands holding one oar, which results in asymmetric shoulder positioning and a different muscle activation pattern between the right and left sides of the torso. This is also very disadvantageous in terms of the loads accumulating in the lumbar spine. Canoeists, however, row only from the side of the lower limb, kneeling^24^. It is speculated that this position may cause differences in tendon extensibility between the kneeling leg and the leg extended forward. The propulsive force of the boat is achieved by lifting the upper body while twisting the trunk muscles and hip joints^25^. Thus, the musculoskeletal load in the case of the canoeist is fundamentally different from that of the rower in that it is distributed virtually across the entire body, whereas in rowers it is the knee joints and primarily the lumbar spine that are loaded.

The optimal dose of physical activity is important to maintain the proper balance in turnover of the cartilage tissue. It has been shown that exercise can be both beneficial and detrimental to articular cartilage health, however, suitable dose (magnitude, duration, and frequency) of exercise may differentiate these effects^6^. On the other hand, repetitive impact and loading in athletes may cause articular cartilage damage. Mechanical stress or traumatic joint injury are mentioned, among others, as risk factors associated with OA^26^. Therefore, it may be speculated that the higher serum concentration of COMP in rowers compared to canoeists probably indicates − given the difference in musculoskeletal load − that these athletes are more susceptible to the adverse consequences of articular cartilage changes associated with sports training.

Amoako et al.^27^ in their review suggested that within the athletic population, beyond genetic conditions, factors such as body mass and muscle strength also contribute to the susceptibility of joints to injuries. In their review, Mazor et al.^6^ suggested that resistance training may cause cartilage degradation and increase OA, however, increasing muscle strength gives the joints greater support and strengthening muscles is one of the fundamental ways to help protect joints. The correlations observed in our study in the entire group of the tested athletes may correspond to the suggestions of the aforementioned authors. Indeed, numerous significant correlations were noted between COMP and aggrecan concentrations and biomechanical parameters, which may be indicative of the involvement of muscle strength in the articular cartilage response to mechanical stimuli. There is, for example, a negative correlation between COMP and the trunk flexor-to-extensor ratio and, at the same time, a negative relationship with the left hip flexor-to-extensor ratio and a positive relationship with the global strength of the right lower limb. These relationships are justified by loading of the athlete's osteoarticular system at the start of the oar stroke phase, in which the hip joints are maximally flexed with the knee joints and the trunk flexed at the same time. Thus, this is the moment of the rowing cycle when the flexor muscles are loaded to the maximum. This contributes to their contracture which progresses over time, thereby leading to greater compression of the aforementioned joints and the intervertebral disc in the L1–S1 spinal region. This most probably affects the metabolism/degradation of cartilage tissue as evidenced by the COMP concentration.

In the case of aggrecan, on the other hand, which, being a proteoglycan, gives articular cartilage the ability to withstand compressive loads^5,7^. Such loads occur primarily in intervertebral discs which are particularly loaded in the case of rowers. In our study, we found a tendency towards a considerably negative relationship between this marker and TPT-E, which also act as active stabilisers of the lumbar spine^26^. A similar relationship was also noted for the EJPT-LF. In contrast, there is a significant relationship between the aggrecan level and the EJPT-RF and EJPT-RE. In this joint the flexor muscle is stronger than the extensor. Independently of the flexion movement, it also performs an inhibitory function on extension movement caused by the forearm gravity torque^28^. However, in canoeists, it is the triceps brachii muscle that plays an important role in movement, with its peak torque considerably higher for both the left and right upper limb simultaneously, compared to rowers. Furthermore, this creates a pathogenic imbalance between the flexor and extensor muscles. This, in turn, results in greater compression of the joint, which contributes to a reduction in the strength of the cartilaginous joint structures over time, manifesting itself in increased aggrecan levels.

The observed correlation between serum COMP levels and the training seniority of canoeists may indicate that the articular cartilage is more susceptible to training loads in more advanced athletes. It is also worth noting that there is a correlation between COMP concentration in the entire group of athletes studied (n = 39) and body height, which confirms the concept of the dependence of the level of this indicator on the impact of mechanical loads on joints, in this case related to body height. The taller the athlete, the longer the lever arms of gravity of the elements comprising each joint. According to the pathomechanics of the musculoskeletal system, the main factor causing its overload changes while assuming various body positions is the gravity torque of the mobile parts forming a particular joint, which, according to its definition, increases together with an increase in the lever arm length.

In this study, we assessed the concentration of 25(OH)D, an indicator of the body's supply of vitamin D (both from food intake and cutaneous synthesis)^29^. In a study conducted on a large group of athletes (outdoor and indoor athletes), Krzywanski et al.^30^ showed that the level of 25(OH)D in athletes training outdoors was significantly higher at different times of the year than in indoor athletes. The study involving rowers and canoeists took place in autumn, however, a considerable influence of environmental factors (exposure of the skin to UV radiation) on the level of this indicator in the period preceding the study cannot be excluded. This phenomenon may be confirmed by the positive correlation of serum 25(OH)D levels and body height in the entire group of athletes (n = 39) and a similar correlation of this indicator with body mass and height in the group of canoeists, which may suggest a dependence of cutaneous vitamin D synthesis on body surface area. In the animal study^31^, authors observed the effects of vitamin D deficiency on articular cartilage degradation by testing matrix metalloproteinase activities in articular cartilage. In our study no correlations were noted between 25(OH)D concentrations and rates of articular cartilage degradation in rowers and canoeists.

The mechanism of action of vitamin D on muscle strength is also known^32^. In our study, significant or tendencies towards relationships between 25(OH)D concentrations and muscle function, according to biomechanical tests, were reported. It should be emphasised that the above correlations primarily concern the muscle groups that perform most of the work during the rowing cycle. Based on the research described in the review paper by Latham et al.^33^ it may be speculated that these correlations are related to the involvement of vitamin D in the regeneration of damaged muscle fibres.

The main limitation of this study was the number of participants. Only high-class athletes were qualified for the tests, so it was difficult to select a larger group of respondents. Another limitation of the study was presentation of biochemical indices only at rest; it would be interesting to analyse the response of cartilage metabolism markers also under exercise conditions. Furthermore, it would be interesting to assess the changes of 25(OH)D in the studied athletes during the entire training cycle.

Based on the results of the study and taking into account previous reports about adverse consequences of articular cartilage overload changes associated with sports training, there are some important considerations. The numerous correlations of cartilage metabolism markers levels with peak torques of the studied muscle groups in rowers and canoeists are indicative of a significant importance of stabilisation of the muscular system in cartilage metabolism. Therefore, in the prevention of adverse changes to the articular cartilage, a set of exercises that reduce the imbalance between the flexor and extensor muscles should be used systematically in the training of athletes. Moreover, relationships between serum 25(OH)D levels and biomechanical parameters confirm the need for controlling vitamin D status in the winter period and possibly to provide athletes with an adequate supply of this vitamin to athletes for the regeneration of skeletal muscle subjected to training loads.

## Methods

### Participants

The study included 24 male sweep-oar rowers and 15 canoeists, members of the polish national teams. Participants declared that they had no active infections, did not take anti-inflammatory drugs and that they fulfilled the conditions of the study protocol (e.g. fasting). Five rowers declared vitamin D supplementation in the period from a few days to 3 months preceding the study, therefore, an additional comparative analysis for aggrecan, COMP and 25(OH)D between both groups of athletes was conducted after excluding these rowers. However, this analysis did not significantly change the results and these rowers were finally included in the statistical analyses of the study results.

The study was carried out at the beginning of the preparatory period (November). A detailed description of the training sessions can be found in an earlier publication^34^.

### Ethics approval and consent to participate

The study was approved by the Regional Bioethics Committee at Poznan University of Medical Sciences (No. 208/17). Each of the athletes expressed a written consent to participate in the study. All methods were performed in accordance with the relevant guidelines and regulations.

### Biochemical analysis

Blood samples were taken fasting between 7.00 and 8.00 a.m. from the ulnar vein, then centrifuged (1500 g, 4 °C) in order to separate the serum. The samples were frozen and stored at − 70 °C until the time the analyses were performed. The immunoenzymatic enzyme-linked immunosorbent assay (ELISA) method was used to measure concentrations of the human COMP (BioVendor, Brno, Czech Republic, sensitivity 0.4 ng/ml, intra- and interassay variability were 4.0–8.0% and 3.1–6.6%, respectively) and human aggrecan (PG, Brno, Czech Republic, sensitivity 0.9 ng/ml, intra- and interassay variability were 1.9–2.6% and 6.05–6.5%, respectively). The concentration of 25(OH)D was determined by electrochemiluminescence immunoassay (ECLIA) method (Roche, Elecsys Vitamin D3 III, sensitivity 3 ng/ml, laboratory intra- and interassay variability were 0.5–1.51% and 4.4–5.81%, respectively).

### Biomechanical measurement procedures

Peak torque (PT) measurements under isokinetic conditions of trunk rotators, trunk flexors and extensors, and upper and lower limb joints were performed using three separate devices manufactured by JBA Zbigniew Staniak (Poland).

**Figure 3:**
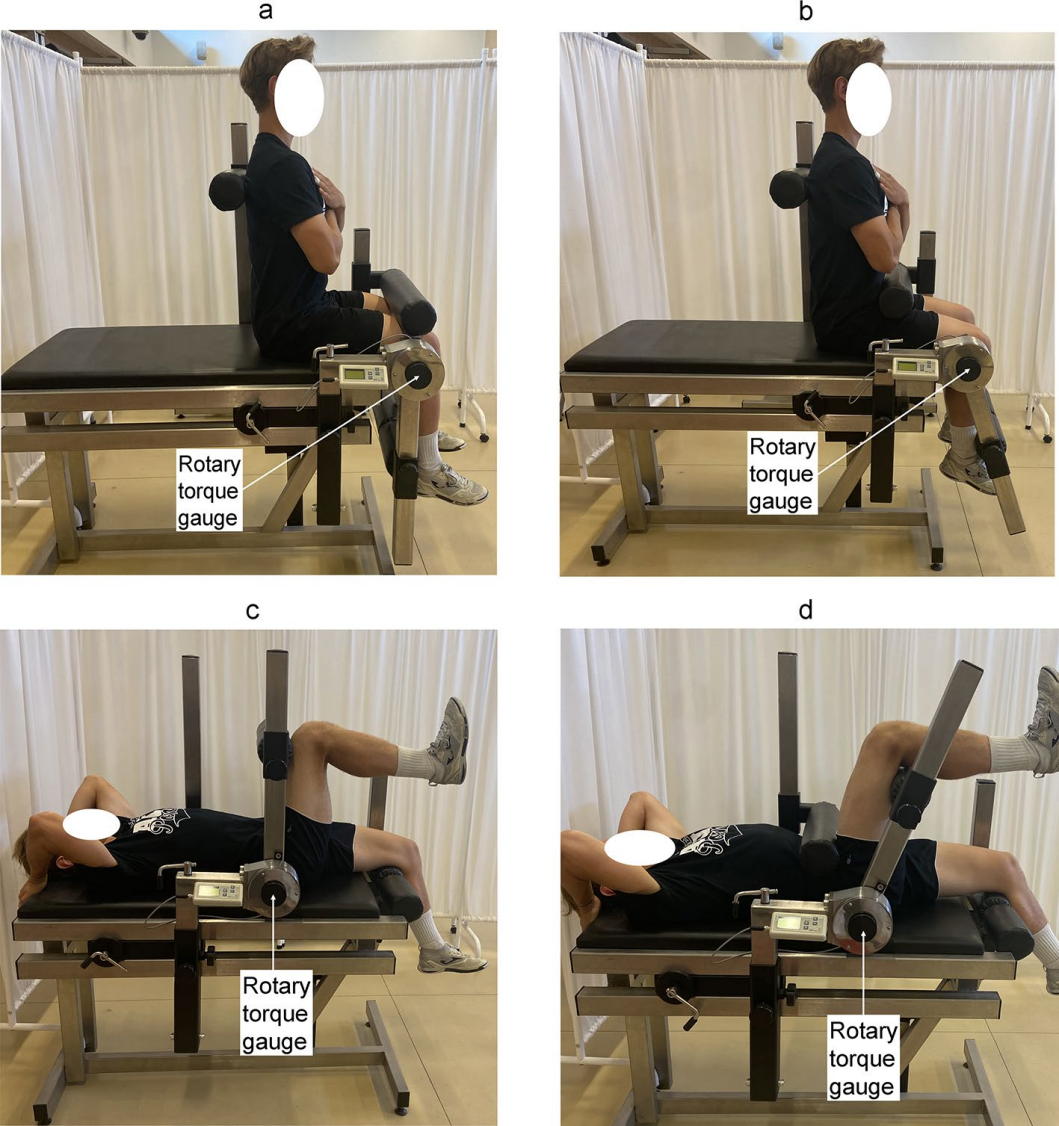
Measurement equipment of the flexors and extensors torque: knee joint (a,b) in the sitting position and hip joint (c,d) in the supine position. The characteristic angles of the torso-thigh and the thigh-shank are 90°. The methodology described by Straburzyńska et al.^35^ https://creativecommons.org/publicdomain/zero/1.0/deed.en.

**Figure 4:**
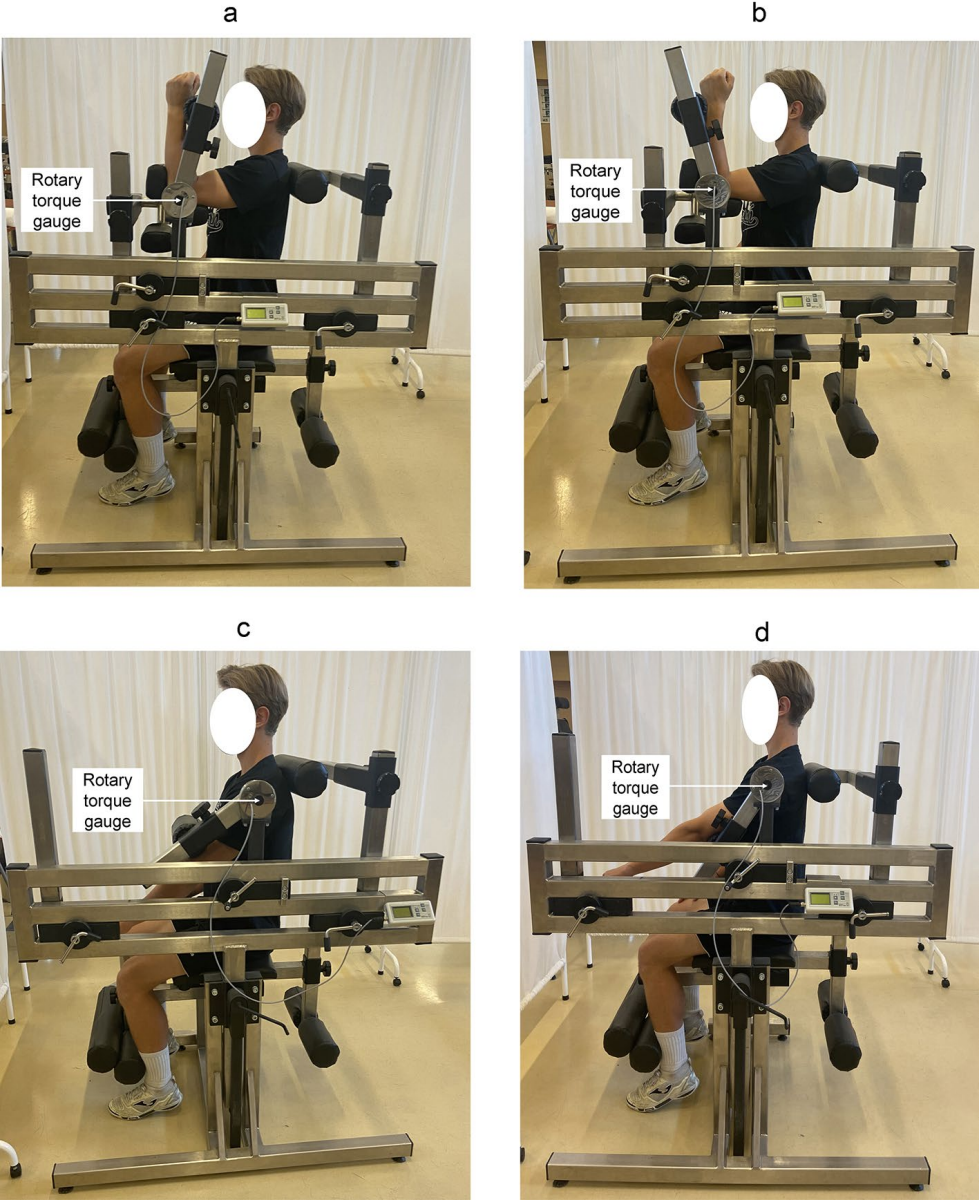
Measurement equipment of the flexors and extensors torque: elbow joint (a,b) and shoulder joint (c,d) in the sitting position. The characteristic angles of the torso-arm and forearm-arm are 90° (a,b), while the torso-arm angle is 45° (c,d).The methodology described by Straburzyńska et al.^35^ https://creativecommons.org/publicdomain/zero/1.0/deed.en.

PT of the knee and hip joints was measured on the TBK3-P station (Fig. 3) in which the measurement range of the torque gauge was 1200 Nm and the maximum measurement error was 1%. Elbow and shoulder joint PT was determined using the LR2-P station (Fig. 4) equipped with a torque gauge with a measurement range of 500 Nm and a maximum measurement error of 0.5%. The axis of the rotary torque gauge coincides with the axis of rotation at the joint (Figs. 3, 4).

All measurements of force moments at the above joints were performed using the asymmetric technique, i.e. alternating measurements at the left and right joints. The maximum muscle force during the measurement was released in 1.5–3.0 s. The subject repeated each type of measurement three times with 30 s intervals, while the intervals between measurements at different joints were approximately 30 min. The highest of the 3 recorded force moment values was used as the final measurement result.

**Figure 5:**
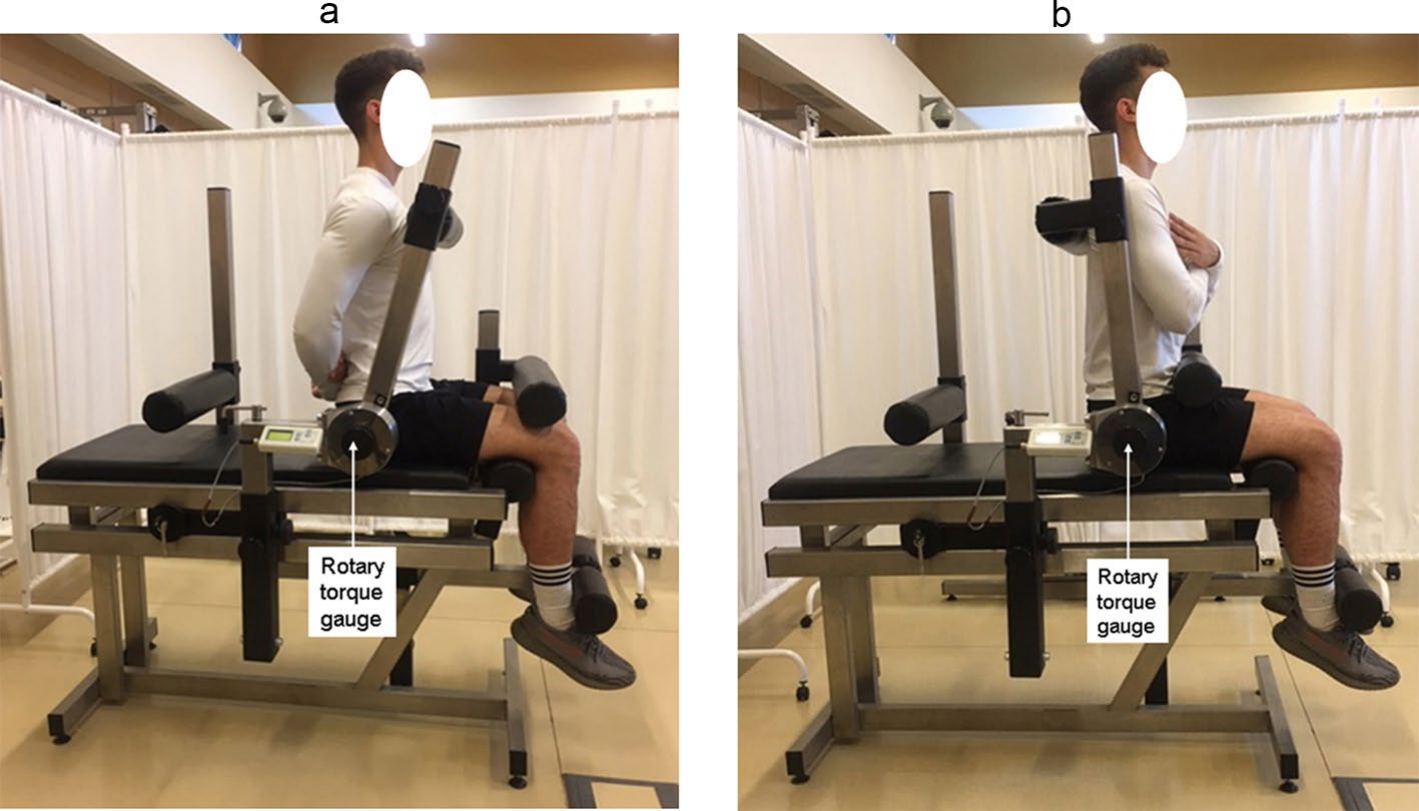
Torque of torso flexors (a) and extensors (b) measurement in a seated position. The methodology described by Podgórski et al.^36^ https://creativecommons.org/publicdomain/zero/1.0/deed.en.

**Figure 6:**
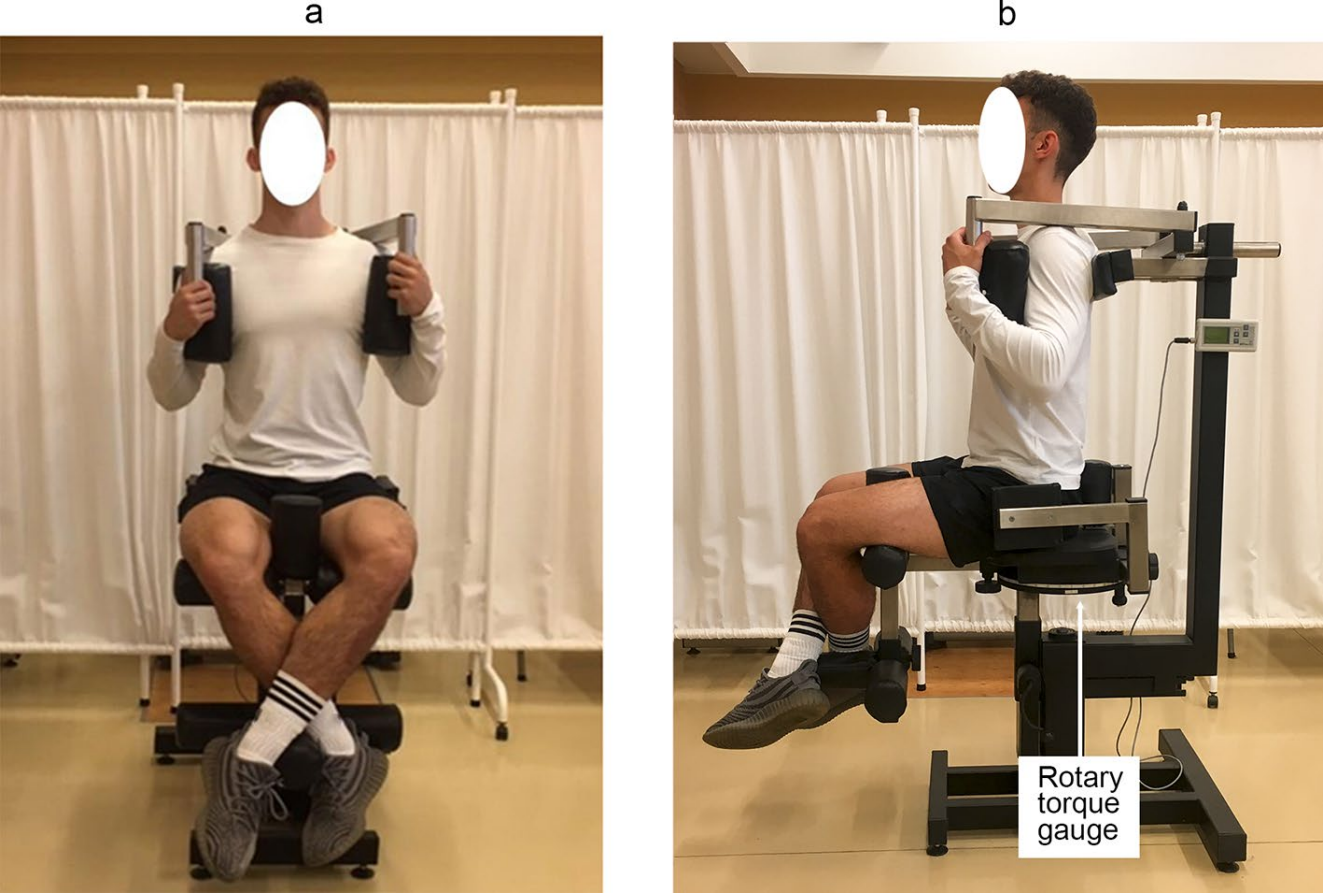
Torque of torso rotators muscles measurement in a seated position in (a) frontal view and (b) sagittal view. The methodology described by Podgórski et al.^36^ https://creativecommons.org/publicdomain/zero/1.0/deed.en.

**Figure 7:**
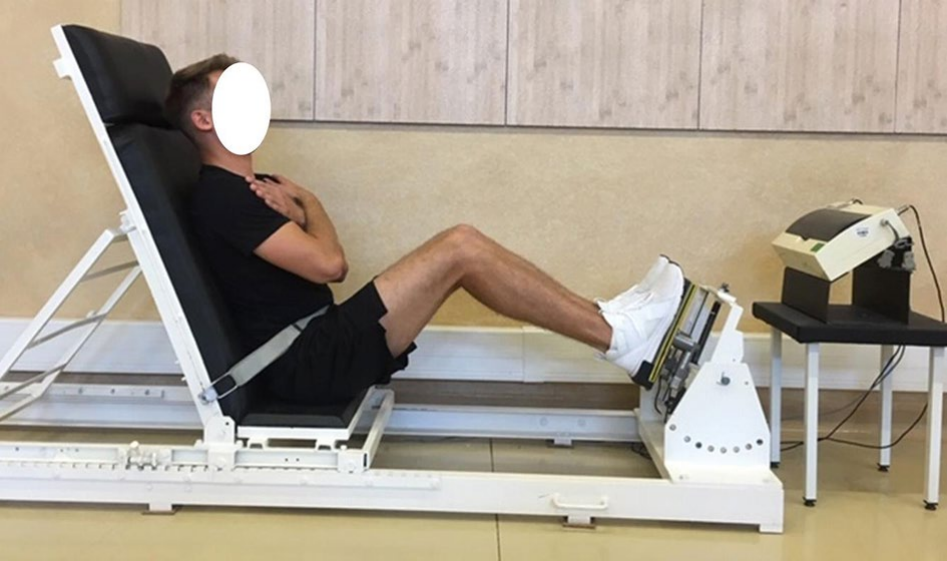
Global force of lower limb extensors measurement in a sitting position. The methodology described by Podgórski et al.^36^ https://creativecommons.org/publicdomain/zero/1.0/deed.en.

Devices equipped with torque gauges with a relative measurement error of less than 0.5% were used to measure the PT of the flexors, extensors and rotators of the trunk (Figs. 5 and 6). In our study, we also investigated global lower limb extension strength (Fig. 7) using a device equipped with the Scaime SB30X dynamometer (relative strength measurement error ± 0.017%) and the PUE 1 m (Radwag®, Poland).

The global force of lower limbs extensors is the resultant force generated simultaneously by extensors of hip, knee and ankle joints. Lower limbs were measured asymmetrically, i.e. alternately left/right limb, respectively.

The construction of the devices ensures optimal stabilization of the limbs and torso and the possibility of taking measurements in body positions which are normally subject to measurement in biomechanical laboratories. The global force of lower limb extensors and torque of flexors, extensors and torso rotators under static conditions triggered in 1.5–3.0 s were recorded. Each type of measurement was repeated 3 times. The final result was the highest of the 3 recorded values of force or torque, normalized to body weight.

### Statistical analysis

The results are presented as mean values, standard deviations (SD), medians and quartile distributions (Q1–Q3). The Shapiro–Wilk test was used to evaluate the normality of distribution. The differences between groups of athletes and normally distributed variables were investigated using the T-test; the U-Mann–Whitney test was used for non-normally distributed variables. Interactions between the discipline, body mass and body height for the COMP and aggrecan variables were performed using an equal slope model. Spearman’s rank analysis was used to calculate correlation coefficients. The level of statistical significance was set at *p* < 0.05. The obtained results were analysed using the Dell Inc. (2016) Dell Statistica 13 software (Tulsa, Oklahoma, USA).

## Conclusions

The numerous correlations of COMP and aggrecan levels with peak torques of the studied muscle groups in rowers and canoeists are indicative of a considerable importance of stabilisation of the muscular system in cartilage metabolism. The higher serum COMP levels in rowers compared to canoeists may indicate increased levels of cartilage tissue metabolism/degradation in these athletes, which are related to the specific biomechanics of rowing. The correlations between 25(OH)D status and biomechanical parameters confirm that vitamin D plays an important role in maintaining skeletal muscle health.

## Data Availability

The datasets generated during and/or analyzed during this study are available from the corresponding author on reasonable request.
